# Electroactive composite biofilms integrating Kombucha, Chlorella and synthetic proteinoid Proto–Brains

**DOI:** 10.1098/rsos.240238

**Published:** 2024-05-29

**Authors:** Anna Nikolaidou, Panagiotis Mougkogiannis, Andrew Adamatzky

**Affiliations:** ^1^ Unconventional Computing Laboratory, University of the West of England, Bristol, UK; ^2^ School of Architecture and Environment, University of the West of England, Bristol, UK

**Keywords:** thermal proteins, proteinoids, unconventional computing, Kombucha cellulose biofilms, memristors, soft robotics

## Abstract

In this study, we present electroactive biofilms made from a combination of Kombucha zoogleal mats and thermal proteinoids. These biofilms have potential applications in unconventional computing and robotic skin. Proteinoids are synthesized by thermally polymerizing amino acids, resulting in the formation of synthetic protocells that display electrical signalling similar to neurons. By incorporating proteinoids into Kombucha zoogleal cellulose mats, hydrogel biofilms can be created that have the ability to efficiently transfer charges, perform sensory transduction and undergo processing. We conducted a study on the memfractance and memristance behaviours of composite biofilms, showcasing their capacity to carry out unconventional computing operations. The porous nanostructure and electroactivity of the biofilm create a biocompatible interface that can be used to record and stimulate neuronal networks. In addition to *in vitro* neuronal interfaces, these soft electroactive biofilms show potential as components for bioinspired robotics, smart wearables, unconventional computing devices and adaptive biorobotic systems. Kombucha-proteinoids composite films are a highly customizable material that can be synthesized to suit specific needs. These films belong to a unique category of ‘living’ materials, as they have the ability to support cellular systems and improve bioelectronic functionality. This makes them an exciting prospect in various applications. Ongoing efforts are currently being directed towards enhancing the compositional tuning of conductivity, signal processing and integration within hybrid bioelectronic circuits.

## 1. Introduction

There is a growing interest in using electrically conductive biofilms for applications that involve the interaction between electronic devices and biological tissues. Conductive biofilms, which consist of microbial communities and biopolymers, are a highly appealing choice for coating materials in implantable electrodes and biosensors. This is primarily due to their biocompatibility and sustainability, which make them superior to synthetic polymers [1,2]. These electroactive biofilms can function as flexible, adaptable coatings that transmit signals between electronic components and electrically excited cells. In microbial fuel cells, for instance, the natural redox and conductive properties of microbial biofilms have been used to improve the power output [3,4]. The incorporation of conductive biocomponents in the development of synthetic bio-electronic biofilms has the potential to facilitate the creation of advanced hybrid bioelectronic systems that can interface with both living and non-living elements.

Thermal proteinoids are abiotic polypeptides that were initially synthesized by Sidney Fox in early 1970s. They are formed by heating mixtures of amino acids, which leads to the formation of sequences that are not directly coded by genes [5]. Proteinoids possess electrical conductivity and memristive properties that can be used in the field of bioelectronics [6,7]. Furthermore, proteinoids have the ability to self-assemble into microspheres that resemble cells, which makes them a fascinating material for bioinspired engineering [8–10].

Kombucha is a beverage made by the process of fermentation, when a symbiotic culture of bacteria and yeasts (SCOBY) is introduced into tea [11]. The tea components are metabolized by the microbial community, resulting in the production of organic acids and a biofilm made of cellulose [12]. Bacteria and yeasts lead a symbiotic life in the cellulosic film layer [13] which increases in thickness as fermentation progresses, providing the necessary oxygen for microorganisms [14]. Microbial metabolism has been used in microbial fuel cells development for electron generation and transfer [15]. For the Kombucha biofilm-forming process, the cellulose serves as an immobilization matrix, resulting in enhanced electron transfer [16].

In this study, we have combined the bioelectronic properties of proteinoids with the matrix-forming ability of Kombucha. Electrically conductive and flexible biocomposite films were created by combining proteinoids and Kombucha. The hybrid material displayed enhanced mechanical strength and electrochemical responses in comparison to the separate components, indicating the presence of synergistic interactions.

The mixture of proteinoids with Kombucha results in a composite material that exhibits unique electrical properties derived from proteinoids, while also benefiting from the matrix-forming, self-growing and self-repairing capabilities of the Kombucha biofilm. The combination of proteinoids and Kombucha in this composite material forms a biofilm that exhibits electrical conductivity, making it a promising candidate for bioelectronic interfaces.

Proteinoids play a significant role in enabling signal transmission through their memristive, memcapacitive and conductive properties. In the interim, the Kombucha culture synthesizes a cellulose membrane that serves as a flexible and cohesive framework for immobilization. Each separate component contributes complementing advantages, resulting in the production of an electroactive biofilm that surpasses the capabilities of each component on its own.

The application of conductive biofilms has great promise in the field of neuronal tissues. The hydrogel-based matrix has favourable characteristics for the application of uniform coating on implantable electrodes, facilitating efficient transmission of signals between electronic devices and excitable cells without interruption. The application of composites presents a viable and environmentally sustainable option for electrode coating, in contrast to the use of synthetic polymers. Moreover, the inherent biocompatibility has the potential to enhance the integration of neurons with electrodes and contribute to sustained performance over an extended period of time.

To summarize, the combination of proteinoids’ distinct bioelectronic properties with Kombucha’s ability to form biofilms shows great potential for creating adaptable conductive interfaces between living and non-living systems. The combination of the two biocomponents has the potential to facilitate the development of sophisticated hybrid bioelectronic devices.

Proteinoids were synthesized through the process of thermal polymerization of amino acids and subsequently introduced into Kombucha during the fermentation process, resulting in the formation of biofilms with conductive properties.

The objectives were:

— To maximize biofilm conductivity and electrochemical activity, it is important to optimize the proteinoid composition and fermentation parameters.
— Analyse various electrical behaviours such as I–V sweeps, transfer functions, coulometry, capacitance and impedance.
— To showcase the proof-of-concept of neuronal interfacing, we will coat microelectrodes and measure the signalling activity using model neurons.

Our hypothesis proposed that the composite material would exhibit a synergistic effect by combining the complimentary advantages of its components, hence facilitating improved communication between neurons and electrodes. The present work aims to provide a comprehensive understanding of the processes involved in biofilm formation, as well as the electrochemical properties associated with it. Additionally, the study explores the potential of using biofilms for interacting with neurons.

## 2. Methods

A Kombucha mat (SCOBY) initially obtained from Freshly Fermented Ltd (Lee-on-the-Solent, PO13 9FU, UK) was used for the production of the Kombucha-proteinoid biofilms. We prepared an infusion of 5 l of boiled tap water, 500 g of white granulated sugar (Tate & Lyle, UK) and 10 tea bags (Taylors Yorkshire Teabags 125 g, UK) in a plastic container. After the solution reached room temperature, the Kombucha mat was placed in the container and stored at 20−23°C in the absence of light. The solution was changed every 10 days and monitored for the production of daughter Kombucha mats. When ready, two daughter mats were transferred from the container and the samples were placed in 12 cm diameter Petri dishes with 20 ml solution each. To enhance the functional properties of the Kombucha biofilm, 10 ml of Chlorella Vulgaris (Blades Biological Ltd) were added to the solution of one sample. Chlorella Vulgaris is a microalgae with a spherical microscopic cell of 2−10 μm diameter [17–19]. We selected Chlorella Vulgaris due to its robustness to changing environmental conditions, fast growth rate [20], its ability to photosynthesize [21] and the presence of proteins in its composition which represents 42–58% of the biomass dry weight [22–26] with 30% migrating in and out of the cell [27]. In addition to this, it has already been demonstrated that Chlorella Vulgaris can act as living biosensor media by exhibiting periodic electrical activity in the form of spikes [28]. The biofilms were kept in the Petri dishes for a week. The proteinoids used in this study were synthesized using the thermal condensation protocol that was previously described in [29]. In this experiment, the amino acids L-glutamic acid and L-arginine were used. These amino acids were purchased from Sigma Aldrich and had a purity level of over 99%. To begin, the amino acids were heated to a temperature of 180°C for a duration of 60 min. This process resulted in the formation of a homogeneous mixture consisting of polypeptide chains. Afterwards, the mixture was dissolved in water to achieve a concentration of 10 mg ml^−1^. The proteinoids were dried using lyophilization. The proteinoids that were obtained were used without undergoing any additional purification steps. A 3 × 3 cm^2^ section of the biofilms was used for the production of the kombucha-proteinoid (KP) biofilm. Two millilitre proteinoid solution were injected in the kombucha biofilm and applied to the surface. The biofilm was then placed back to the Petri dish for 24 h.

**Figure 1.**
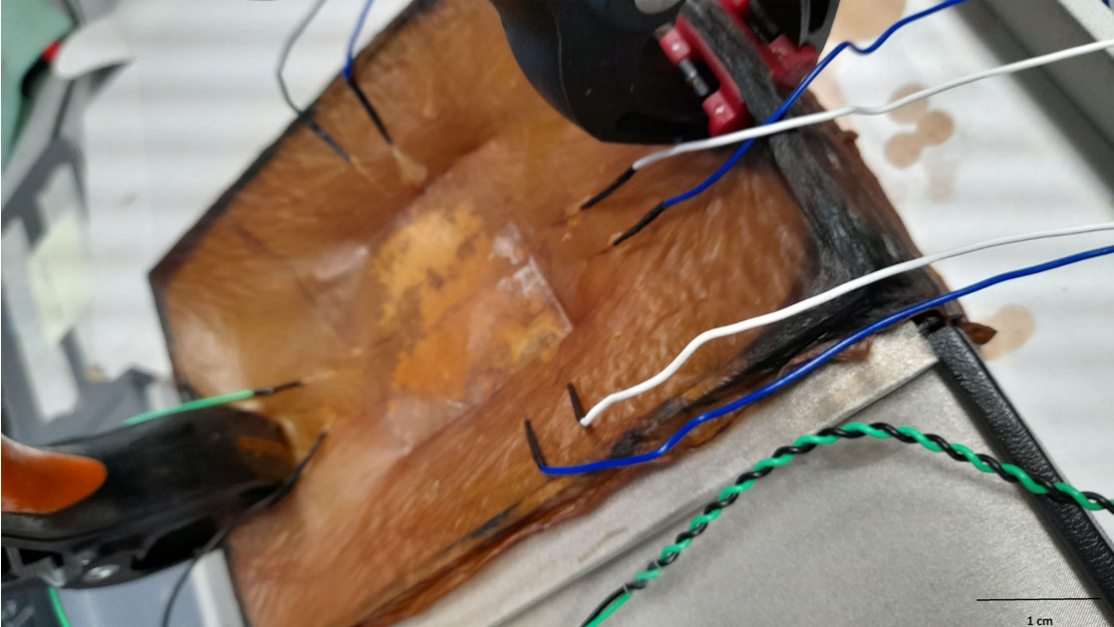
The experimental setup employed for the analysis of electrical spikes in Kombucha using Picolog electrodes. The growth of the Kombucha biofilm proceeds in a sugared tea infusion enriched with the necessary nutrients. Platinum–irhidium wire electrodes are inserted into the Kombucha matrix. The electrodes are linked to a Picolog ADC-24 data logger with high impedance capabilities in order to facilitate the capture and storage of electrical signals. The photograph given depicts a magnified perspective wherein the working electrodes are observed to be entering the Kombucha sample. The experimental configuration enables the application of stimulation to the Kombucha culture using the Picolog hardware, while simultaneously measuring the dynamic variations in current and the characteristics of spikes, which serve as indicators of the electrophysiological activity of the biofilm. The scale bar in the image represents a length of 1 cm, providing a reference for the size of the experimental setup and the Kombucha biofilm sample is in a wet state.

Various instruments were employed to perform dynamic current–voltage (I–V) profiling, impedance, capacitance and resistance measurements for the electrical evaluation of the proteinoid-microbial composite samples. The experimental configuration consisted of platinum-iridium wire electrodes, which were positioned at a distance of 10 mm from each other. These electrodes were in direct contact with the sample material, allowing for the application of voltages and the measurement of currents. An LCR meter (Model 891, BK) was used to conduct impedance spectroscopy within the frequency range of 1 kHz to 300 kHz. The experimental setup involved doing I–V sweep measurements using a Keithley 2450 source meter, where the voltage range varied from −1 V to +1 V. The BK Precision 4053B 10 MHz dual channel function generator was used to supply input waveforms for the purpose of conducting experiments on frequency-dependent capacitance and resistance. The measurements were conducted at ambient temperature with specialized test setups designed to facilitate the connection between the samples and the electrode probes. We used a Picolog ADC-24 high impedance data logger (figure 1) to analyse the electrical activity of the kombucha biofilms. The proteinoid-microbial samples that have been obtained were subjected to characterization using scanning electron microscopy (SEM) with an FEI Quanta 650 field emission SEM.

## 3. Results

### 3.1. Structural characterization of Kombucha-proteinoids complexes by SEM

**Figure 2.**
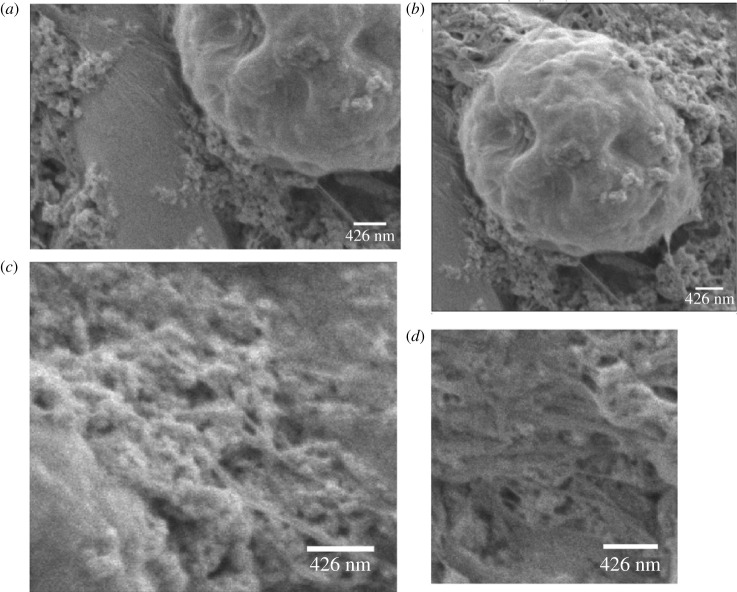
A SEM analysis of the integrated KPs system. (*a*) SEM image of yeast with a diameter of 1394 nm in close proximity to a Chlorella microsphere (4.76 μm in diameter) surrounded by L-Glu:L-Arg proteinoids with a diameter of 104.6 nm. (*b*) SEM image of a complete microsphere of Chlorella. (*c*) The SEM image depicts proteinoids nanospheres. (*d*) A SEM image depicting yeast surrounded by proteinoids nanospheres. All SEM images were captured at 2.00 kV, x27 234 magnification, with a scale bar of 426 nm.

SEM was employed to analyse the structural properties of the integrated system comprising Kombucha and proteinoids. The analysis of the data depicted in figure 2 reveals the presence of yeast cells measuring around 1.4 μm in diameter in close proximity to bigger Chlorella microspheres with a diameter of 4.76 μm. These microspheres are encompassed by proteinoids nanospheres measuring 105 nm in diameter, which are made of L-Glu and L-Arg. Furthermore, it was observed that proteinoid nanospheres were distributed uniformly inside the sample, with a particular concentration observed in the vicinity of yeast cells. The findings presented in this study illustrate the effective reorganization of Kombucha with L-Glu:L-Arg proteinoids, resulting in the formation of a composite structural framework that encompasses both microbial and peptide components. The results of the SEM study indicate that the proteinoid nanospheres are not simply located on the surface of the Kombucha biofilm, but rather they are closely integrated with the microbial and microalgal components. The presence of invasive three-dimensional integration of proteinoids inside the architecture of the Kombucha biofilm requires unique molecular recognition and interaction between the proteinoids and the biofilm components.

The observed integration and adherence of proteinoid nanospheres to the Kombucha biofilm architecture, as detected using SEM, is believed to be assisted by several intermolecular interactions. Electrostatic interactions might potentially arise between the charged amino acid residues, such as glutamate and arginine, located in the polypeptide chains of proteinoids, and the many anionic functional groups found in the extracellular polymeric substances (EPS) of the Kombucha matrix, which are rich in polysaccharides [30]. Furthermore, the proteinoids possess non-polar domains that are dispersed throughout their structure. These non-polar domains have the ability to insert themselves into hydrophobic regions found on the microbial cell walls or penetrate into the EPS matrix through hydrophobic interactions [31]. The proteinoid-Kombucha interface exhibits several hydrogen bonding possibilities, which stem from the hydroxyl, carboxyl and amino groups present on both proteinoid and Kombucha components. In addition, weak van der Waals forces can also contribute to the interactions mentioned above by facilitating a general attraction between the proteinoids and the biofilm surface at close range. The proteinoids and morphological features of the biofilm may exhibit conformational compatibility, which can enhance multivalent bindings and promote increased surface networking [32]. The close association between proteinoids and Kombucha, as discovered in this study, suggests the potential for creating a bio-abio hybrid system that can emulate certain functions of the brain, thereby forming a basic ‘Kombucha-proto brain.’ Proteinoid nanonetworks possess the capacity to function as distributed computing components similar to neurons, facilitating the exchange of impulses throughout a linked polysaccharide matrix facilitated by the Kombucha. The cooperative biomolecular network integrates the information processing and signalling capacities of proteinoids with the structural scaffolding provided by microbial cellulose. Similar to the emergence of the brain, which originated from ancient biochemical transformations such as lipid membranes and protein ion channels, the theory of the ‘Kombucha-proto brain’ uses the self-assembly of abiotic peptides and microbial fluids to imitate the complex processes of excitation, conduction and cognition. The reported bioelectronic functionalities, adaptivity and learning capacities exhibited by ‘Kombucha-proto brains’ embody fundamental characteristics essential for the development of primitive synthetic brains and bio-nervous systems. The continued development of this process of biosynthetic integration could potentially facilitate the development of engineered living materials that possess cognitive abilities, adaptive characteristics and computational capabilities [33–37].

The diverse morphologies seen using SEM offer visual validation of the production of biofilms composed of KPs, exhibiting distinct structural organization. Additional investigation is necessary to thoroughly assess the potential of these integrated biofilms for applications involving neural interface, specifically in terms of their electrical conductivity and stimulatory capacity.

### 3.2. Elucidating the dynamic behaviour of Kombucha-proteinoids networks using sinusoidal inputs

**Figure 3.**
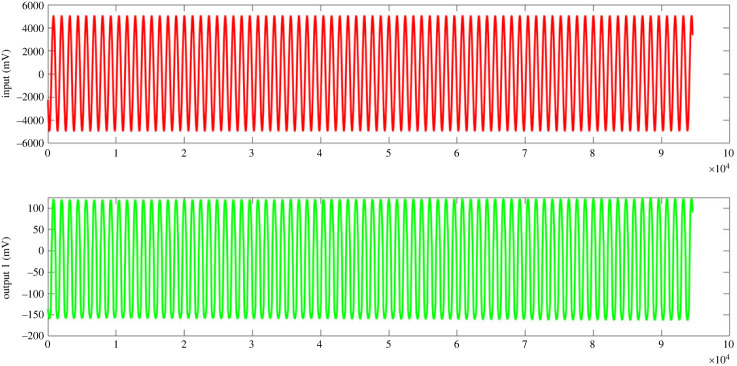
Response characteristics of L-Glu:L-Arg proteinoids. The proteinoids were subjected to an input signal with an amplitude of 5.05 mV and a frequency of 0.056 Hz. (*b*) Signal recorded from the proteinoids as a result of the input signal. Compared to the input values, the measured output signal exhibited a significant decrease in amplitude, measuring 288.55 mV, and a corresponding shift in frequency, measuring 0.119 Hz.

**Figure 4.**
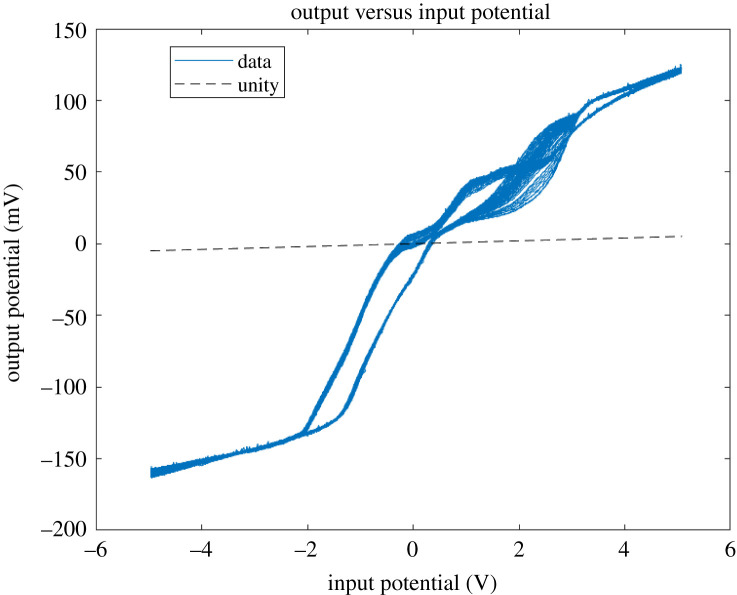
Potential output versus input for the proteinoids system. The output potential of the proteinoids is compared to the input potential of the system. The dashed unity line indicates an input-to-output ratio of one-to-one. The proteinoids’ nonlinear response suggests that they have amplitude-dependent filtering properties.

**Figure 5.**
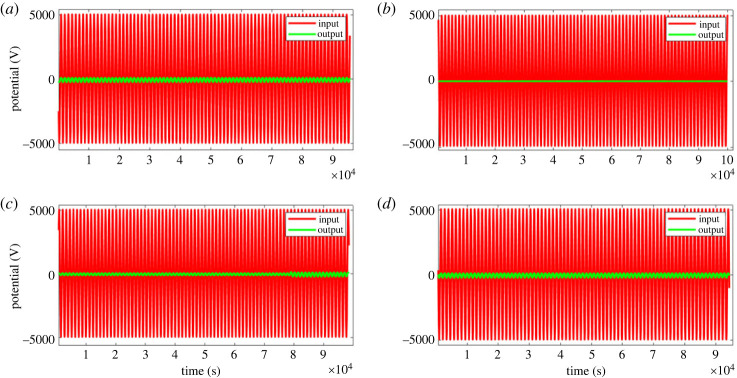
Response characteristics of proteinoids-Kombucha biofilms. (*a*) Sample A with 40% proteinoids, 60% KC showed input amplitude of 10.04 mV, input frequency of 0.055 Hz, output amplitude of 352.28 mV and output frequency of 0.114 Hz. (*b*) Sample B with 40% proteinoids, 60% Kombucha exhibited input amplitude of 9.96 mV, input frequency of 0.055 Hz, output amplitude of 26.13 mV and output frequency of 0.256 Hz. (*c*) Sample C with 25% proteinoids, 75% KC displayed input amplitude of 10.06 mV, input frequency of 0.055 Hz, output amplitude of 360.59 mV and output frequency of 0.120 Hz. (*d*) Sample D with 25% proteinoids, 75% Kombucha showed input amplitude of 9.98 mV, input frequency of 0.056 Hz, output amplitude of 27.41 mV and output frequency of 0.272 Hz. The results demonstrate tuning of the output response based on proteinoids-microbial composition, with KC samples showing higher output amplitudes and minor frequency shifts.

**Figure 6.**
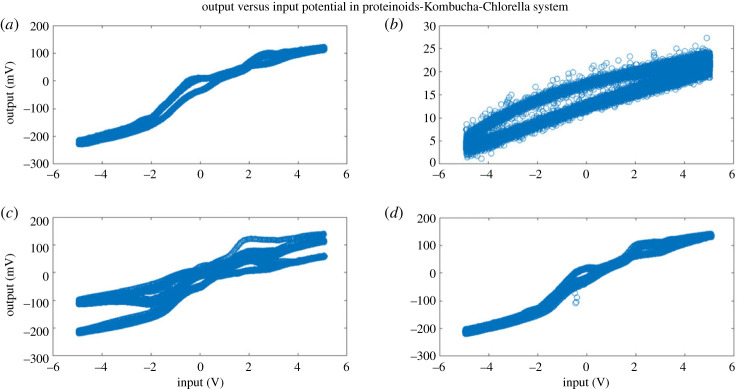
The plot demonstrates the relationship between the input potential and the output potential for four distinct samples of proteinoids-Kombucha biofilms. The samples demonstrate a linear correlation between the two variables. However, it is important to note that the slopes vary depending on the composition of the biofilms. Samples A and C, which contain KC, exhibit steeper slopes compared to samples B and D, which solely contain Kombucha. This implies that samples A and C have the ability to generate higher output potentials than samples B and D, even when given the same input potentials. The plot showcases how proteinoids-Kombucha biofilms can be adjusted to improve signal transmission in various input conditions. This is supported by the observed effects that vary in magnitude depending on the amplitude.

When the proteinoids system was exposed to input signals, it showed a decrease in amplitude along with slight changes in frequency (figure 3). On the other hand, the composites that consisted of proteinoids and Kombucha mixtures exhibited increased output amplitudes and enhanced frequency consistency when compared with the input data (table 1). As an example, after examining sample A composed of 40% proteinoids and 60% Kombucha-Chlorella (KC), it was seen that there was an approximate 35-fold increase in amplitude while the input frequency remained constant. The system consisting solely of proteinoids demonstrated a nonlinear connection between input and output, with a decrease in amplitude observed at higher input amplitudes (figure 4). This indicates the presence of inherent amplitude-dependent filtering capabilities. On the other hand, the proteinoids-Kombucha biofilms exhibited signalling behaviours that were more optimized, as illustrated in figure 5. A linear correlation between input and output was observed for all samples of proteinoids-Kombucha biofilms with samples containing Kombucha and Chlorella generating higher output potentials than samples containing only Kombucha when the same input potentials are applied (figure 6).

**Table 1.** Response characteristics of proteinoids-Kombucha biofilms.

sample	% proteinoids	% Kombucha	input amplitude (mV)	input frequency (Hz)	output amplitude (mV)	output frequency (Hz)
A	40%	60% Kombucha-Chlorella	10.04	0.055	352.28	0.114
B	40%	60% Kombucha	9.96	0.055	26.13	0.256
C	25%	75% Kombucha-Chlorella	10.06	0.055	360.59	0.120
D	25%	75% Kombucha	9.98	0.056	27.41	0.272

The proteinoids-KC composites exhibit enhanced signalling behaviours compared to proteinoids alone, suggesting that there are synergistic interactions taking place between the components of the mixture. The Kombucha and Chlorella are likely to contain a significant amount of hydrophilic polysaccharides and cell surfaces. These elements have the ability to retain higher densities of proteinoids through electrostatic and hydrogen bonding forces. As a result, the conductivity is increased. Furthermore, the cellulosic and microbial nanowires found in Kombucha have the potential to enable the direct connection between neighbouring proteinoid nanostructures. This would establish interconnected pathways for electron transfer throughout the composite network. Chlorella’s endogenous redox proteins and metabolites have the potential to interact with proteinoids, creating novel intramolecular charge transfer pathways. In addition, the three-dimensional matrix formed by the proteinoids, Kombucha and Chlorella components is highly organized. This matrix enhances the connection between nearby domains, allowing signals to be transmitted accurately and effectively. The binding of Kombucha and Chlorella to proteinoids leads to conformational changes, which may lead to the unlocking of alternative conductivity states. These states can have improved transmission efficiency. In summary, the cooperative molecular interactions among the proteinoid, microbial and microalgal constituents seem to work together to enhance electron mobility, charge transfer and conductivity. This allows for the efficient transmission of input signals with amplification.

The findings of this study indicate that the integration of proteinoids with microbial components such as Kombucha modifies the signalling characteristics, resulting in great properties such as increased amplification and a wider range of frequency response. The ability to adjust the input–output relationships allows for the optimization of composition in order to achieve predicted response characteristics. To better understand the signalling behaviours in these integrated biomolecular composites, it is necessary to conduct additional spectroscopic and microscopic experiments. Such research will help us uncover the precise structure-function mechanisms that are responsible for these interactions.

### 3.3. Elucidating the adaptive performance of a ‘Kombucha-Proto Brain’ via multi-frequency impedance and capacitance measurements

**Figure 7.**
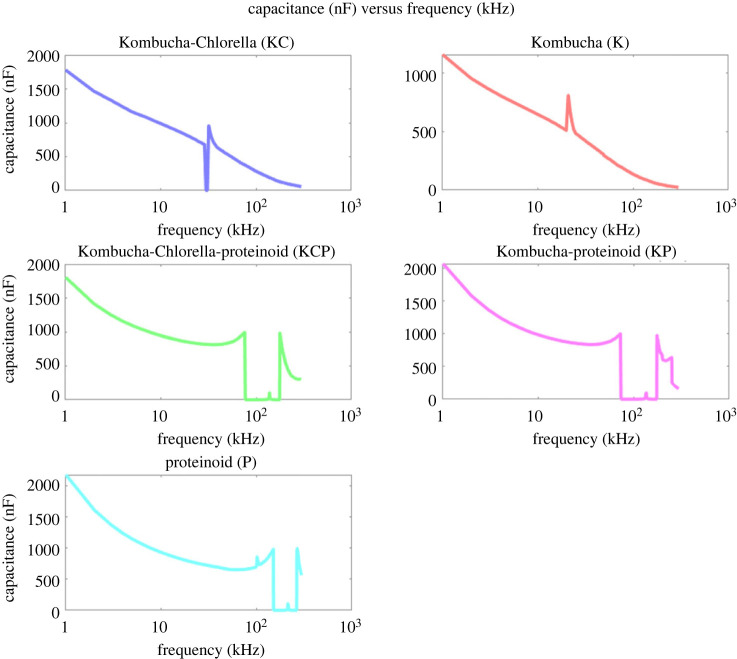
The capacitance as a function of frequency is measured for five different samples: Kombucha-Chlorella (KC), Kombucha (K), Kombucha-Chlorella-proteinoid (KCP), Kombucha-proteinoid (KP) and proteinoid (P). The plot of capacitance is represented on a linear scale, while the frequency is represented on a logarithmic scale. At a frequency of 1 kHz, KC, KCP, KP and P all have a similar initial capacitance of approximately 2000 nF. However, K has a lower starting capacitance of 1150 nF. In general, the capacitance of all samples decreases in a linear manner as the frequency increases. There are some significant deviations from linearity that are worth mentioning. Specifically, there are spikes in capacitance observed for KC at 29 kHz and K at 21 kHz. Additionally, there are capacitance wells observed between 72 and 269 kHz for KCP, KP and P samples. The complex impedance spectra of capacitance demonstrate the presence of frequency-dependence and nonlinear characteristics. This indicates that changes in the microstructures and interfaces within the composite biofilms are influenced by voltage. The capacitance wells observed between 72 and 269 kHz for the KCP, KP and P samples suggest the presence of frequency-dependent and nonlinear characteristics in these composite biofilms. These characteristics are likely influenced by the complex microstructures and interfaces within the biofilms, which can be sensitive to voltage changes. It is important to note that the capacitance drop around 100 Hz is not observed in the KC and K samples, which do not contain proteinoids. This suggests that the presence of proteinoids in the KCP, KP and P samples may contribute to the distinct electrical behaviour and capacitance response at lower frequencies.

Capacitance measurements across a range of input frequencies were implemented to examine the adaptive properties of the KP proto-brain system (figure 7). The composite biofilms exhibited voltage-dependent characteristics in their capacitance profiles, wherein an overall decrease in capacitance was noted as the frequency increased. The observed decrease in capacitance is likely attributed to the influence of dielectric polarization occurring within the microbial and proteinoid components. At higher frequencies, the applied field can induce molecular dipole oscillations which do not fully align, lowering the composite permittivity and thereby the measured capacitance [38–40]. The presence of irregular peaks and troughs observed in the capacitance-frequency graphs indicates the occurrence of distinct channels for charge transfer within the architecture of the proto-brain, which are activated when specific frequency resonances are reached. A variable external input signal causes frequency-dependent capacitance variations in the KP proto brain system. Capacitance modulation in response to varied input frequencies shows that the KP network actively adapts and reconfigures its structure and characteristics dependent on the stimuli. This property is an essential feature of adaptive systems. The composite ‘architecture’ undergoes molecular and morphological adaptations as the input frequency changes. These adaptations result in changes to its dielectric properties and charge storage capacity. The tunable capacitance is likely a result of dynamic polarization mechanisms occurring within the proteinoid nanonetworks and their interfaces with the polysaccharide matrix. The conformation and alignment of proteinoids can be altered at various frequencies, thereby modulating dipolar interactions. In addition, the Kombucha component may experience changes in cell wall permeability, hydration and interior conductivity due to voltage, which collectively affect the overall capacitance of the system. The outcome is a versatile material that has the ability to process information in a ‘context’-dependent manner, similar to natural neural systems [41–45].

**Figure 8.**
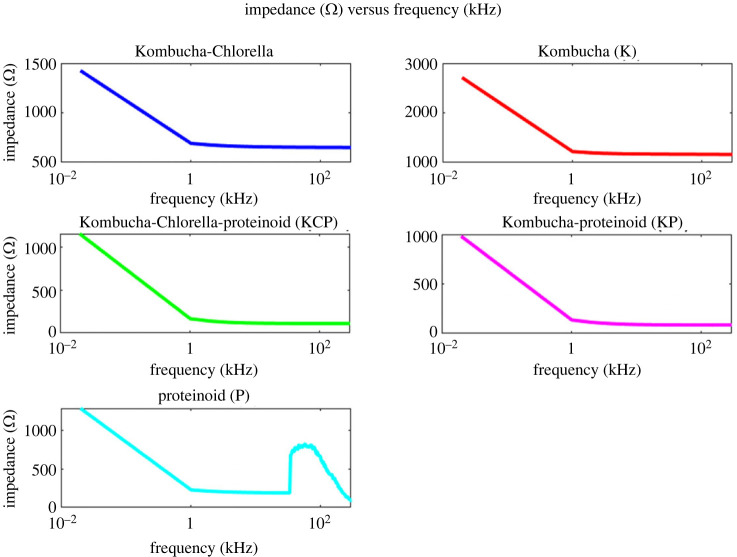
The impedance spectra were obtained for samples of Kombucha-Chlorella (KC), Kombucha (K), Kombucha-Chlorella-proteinoid (KCP), Kombucha-Proteinoid (KP) and Proteinoid (P). At a frequency of 0.02 kHz, the impedance values are as follows: 1425 Ω for KC, 2711 Ω for K, 1150 Ω for KCP, 981.4 Ω for KP and 1285 Ω for P. The impedance decreases in a linear manner until it stabilizes at 1.02 kHz. At this frequency, the impedance values are as follows: 691 Ω (KC), 1220 Ω (K), 165.8 Ω (KCP), 136.5 Ω (KP) and 228 Ω (P). This plateau indicates frequency-independent impedance above 1 kHz. One notable exception is the P sample, which exhibits a significant increase in impedance from 189.5 to 821.1 Ω within the frequency range of 34–278 kHz. The spectra provide insights into passive electrical properties that are dependent on voltage. These properties are likely a result of structural changes occurring at the interfaces of microbial and proteinoid components. Equivalent circuit modelling could determine specific conductive pathways obtained at different input frequencies [46,47].

**Figure 9.**
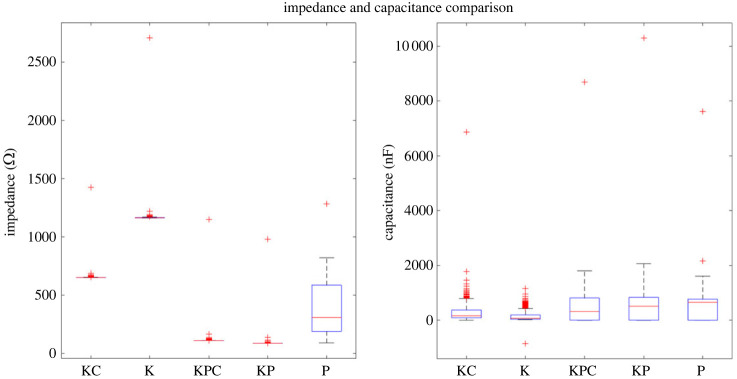
A boxplot comparison is conducted to analyse the impedance and capacitance of different samples, including Kombucha-Chlorella (KC), Kombucha (K), Kombucha-Chlorella-proteinoid (KCP), Kombucha-proteinoid (KP) and proteinoid (P). The box represents the values of the first quartile, median and third quartile, while the whiskers show the variability beyond the upper and lower quartiles. Individual points outside the whiskers represent outliers. The marks on the graph indicate the median confidence interval. The impedance measurement shows that K has the highest median value, while KCP has the lowest spread. In the context of capacitance, the variable P exhibits the highest median value, while KP demonstrates the widest range of variation.

Furthermore, the frequency-dependent impedance of the samples offered valuable insights into their adaptive response, as depicted in figure 8. The impedance spectra mirrored capacitive trends, showing an early decrease followed by the attainment of plateau values. These plateau values suggest the presence of a frequency threshold beyond which the passive electrical properties remain stable. Nevertheless, the proteinoid sample exhibited distinct impedance modulation throughout the frequency range of 34–278 kHz, which corresponds to the reported oscillations in capacitance that are exclusively associated with samples containing proteinoids. The changes in capacitance and impedance that are correlated at specific resonant frequencies indicate the activation of particular conductive pathways and charge transfer processes. The passive electrical characteristics of the proto-brain samples were statistically compared through boxplots of the impedance and capacitance distributions (figure 9).

Table 2 provides a summary of the capacitance measures for each type of sample. An analysis of the median values reveals a discernible pattern of escalating capacitance among the different samples, ranging from Kombucha (K) to proteinoid (P). Notably, the proteinoid sample demonstrates the greatest median capacitance, measuring at 658.6 nF. This suggests that the incorporation of proteinoids with microbial components leads to an enhancement of the dielectric permittivity on a broader scale. Furthermore, it is worth noting that the KPC sample exhibited a substantial range of 8681.994 nF in capacitance, indicating a notable modulation of capacitance in response to voltage. On the other hand, the sample containing just Kombucha (K) exhibited the most limited range of 2009.1 nF. The observed differences in the median and range of values demonstrate the collaborative influence of proteinoid and microbial constituents on the overall dielectric properties at all levels. A noteworthy characteristic observed in the capacitance data is the presence of a low capacitance value of –850.1 nF, which was recorded for the Kombucha (K) sample. The presence of negative capacitance in microbial biofilm networks indicates the occurrence of active voltage-dependent processes. This stems from dynamic feedback mechanisms that counteract the applied AC field through nonlinear molecular conformations or charge injections. The membrane potential of Kombucha is likely to have an effect in conjunction with proton and ion fluxes, which can temporarily reverse the field response [49,50].

**Table 2.** Capacitance statistics for each sample type. The presence of negative capacitance values, particularly in the K sample, suggests the occurrence of phenomena similar to those observed in ZnO thin films [48]. The loss of metal/semiconductor interface charge states under forward bias conditions, along with the presence of oxygen vacancies and piezoelectric/electrostriction effects, may contribute to the negative capacitance behaviour in the composite biofilms. This negative capacitance phenomenon has the potential to be exploited for reducing the limiting effects in the downscaling of electronic devices and going beyond the Boltzmann limit, as demonstrated in ZnO thin-film-based field-effect transistors [48].

sample	median (nF)	min (nF)	max (nF)	spread (nF)
KC	164.7	1.091	6874	774.9
K	73.38	−850.1	1159	2009.1
KPC	325.8	1.006	8683	8681.994
KP	515.3	1.004	10 300	10298.996
P	658.6	1.002	7623	7621.998

Examining the impedance metrics across varieties of samples reveals distinct tendencies (table 3). The Kombucha (K) sample displayed the maximum median impedance of 2655.5 Ω, reflecting greater resistive properties. In contrast, the Kombucha-proteinoid (KP) combination had the lowest median impedance at 945.5 Ω, indicating complexes more conducive to charge transport. Due to conformational changes, the Kombucha (K) sample exhibited the widest spread between minimum and maximum impedance values of 181 Ω. This indicates greater impedance variability. By contrast, KPC had the smallest dispersion of 148 Ω, indicating a more stable and ordered conductive network. The impedance profiles of Kombucha-Chlorella (KC) and Kombucha-Chlorella-proteinoid (KCP) samples were intermediate.

**Table 3.** Impedance statistics for each sample type.

sample	median (Ω)	min (Ω)	max (Ω)	spread (Ω)
KC	1408.5	1256	1510	254
K	2655.5	2500	2820	320
KPC	1102	1050	1198	148
KP	945.5	869	1023	154
P	1215	1120	1301	181

The conductivity profiles of the proto-brain samples, which vary depending on frequency, offer additional insights into their adaptive electrical response characteristics. The conductivity *σ* was determined by using the measured capacitance values and applying the following relation:

3.1 $$\sigma =\frac{1}{\omega C\sqrt{1+\tan^{2}(\phi )}}.$$

**Figure 10.**
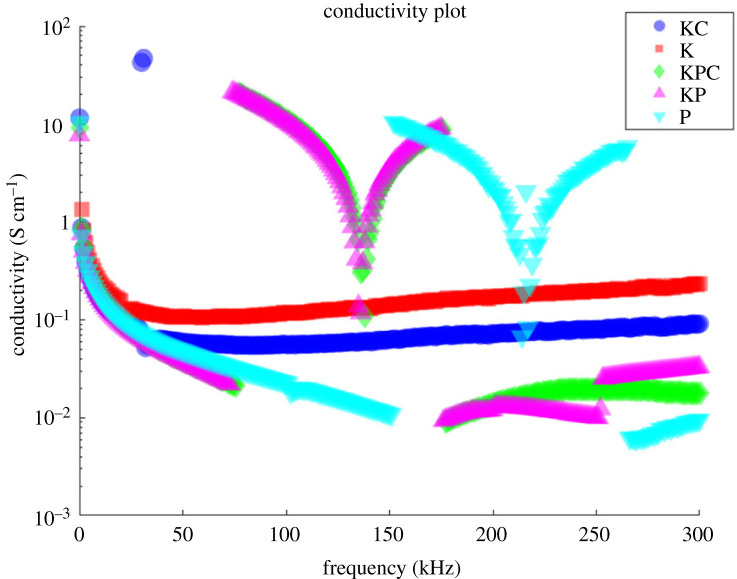
The conductivity profiles for various mixtures of components in Kombucha are as follows: Kombucha-Chlorella (KC), Kombucha (K), Kombucha-Chlorella-proteinoid (KCP), Kombucha-proteinoid (KP) and proteinoid (P). These patterns of conductivity (S cm^−1^) vary depending on the frequency (kHz). The conductivity was obtained by taking the inverse of the capacitance measurements and converting the units. The KC, KPC and P samples exhibited the highest conductivity across a wide range of frequencies, indicating a higher level of charge mobility. The samples showed a decrease in conductivity as the frequency increased. However, the KPC and KP samples showed significant increases in conductivity, with rises of over three orders of magnitude between 74 and 178 kHz. The conductivity rose from 0.03 to 20 S cm^−1^. The P sample exhibited a comparable increase in conductivity within the frequency range of 152–259 kHz. The occurrence of these unusual increases implies that there is activation of enhanced conduction mechanisms at specific frequency resonances. The conductivity spectra demonstrate the adjustable charge transport properties of the composite biofilms. These properties can be specifically controlled by varying the frequency of the input signal.

The symbol *ω* represents the angular frequency, C represents the capacitance and *ϕ* represents the phase angle between the current and voltage. This model takes into consideration both resistive and reactive contributions across a frequency range of 0–300 kHz [6]. The conductivity profiles provide additional information to the capacitive trends, so presenting an in-depth understanding of the proto-brain’s ability to effectively transport charge and reduce signal attenuation. Conductivity is also directly influenced by molecular-level factors including ion mobility, membrane permeability and structural order [51–54]. A closer look at the frequency dependency may thus provide valuable mechanistic insights into the dynamic reorganization of conductive components within the proteinoid-microbe matrix (figure 10). Multi-modal electrical analysis provides a comprehensive understanding of how the emerging proto-brain network adjusts its properties to encode and transmit information.

**Figure 11.**
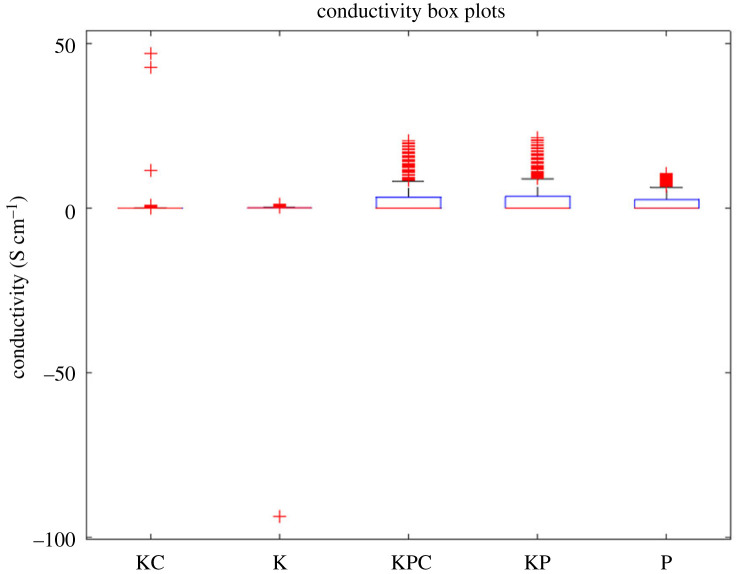
A boxplot comparison is conducted to analyse the conductivity values for five different samples: Kombucha-Chlorella (KC), Kombucha (K), Kombucha-Chlorella-proteinoid (KCP), Kombucha-proteinoid (KP) and proteinoid (P). The K and P samples exhibited the highest median conductivities. The KPC demonstrated the largest difference between the first and third quartiles, which suggests that it has a wide range of tunable conductivity. The KC sample exhibited the highest number of outliers, suggesting that it had greater variability in its conduction properties. In general, the composites that included proteoinoids (KPC, KP) showed higher conductivity when compared with Kombucha alone.

The conductivity metrics for each sample are presented in table 4. Upon examining the median conductivity, it is evident that the Kombucha-proteinoid (KP) and Kombucha-Chlorella (KC) samples displayed the highest values, suggesting a higher degree of charge mobility. The presence of a significantly negative minimum conductivity in the Kombucha (K) sample suggests that there are active processes taking place within the microbial network. Furthermore, the composites of Kombucha-proteinoid (KP), Kombucha-Chlorella-proteinoid (KCP) and Kombucha-Chlorella (KC) exhibited conductivity spreads exceeding 20 S cm^−1^, indicating their ability to achieve a wide range of tunable conductivity. In general, the combination of proteinoids with the microbial components resulted in increased conductivity compared to using Kombucha alone (figure 11). The statistics on conductivity provide insights into how the cooperative interactions between components affect the overall conduction properties of these composite biosystems.

**Table 4.** Conductivity statistics for each sample type. The minimum conductivity value for the K sample is negative. One possible explanation for this outlier is the presence of charge carrier trapping or scattering phenomena at the electrode-sample interface, leading to a temporary reduction in the measured current and a consequent negative conductivity value.

sample	median (S cm^−1^)	min (S cm^−1^)	max (S cm^−1^)	spread (S cm^−1^)
KC	0.069348	0.051857	47.027684	46.975827
K	0.156378	−93.609542	1.346284	94.955825
KPC	0.038001	0.009141	20.543528	20.534387
KP	0.038905	0.009258	21.288726	21.279469
P	0.063023	0.005958	10.449820	10.443863

### 3.4. Realization of logic gates through signal mixing and thresholding in Proteinoids-Kombucha-Chlorella biofilms

**Figure 12.**
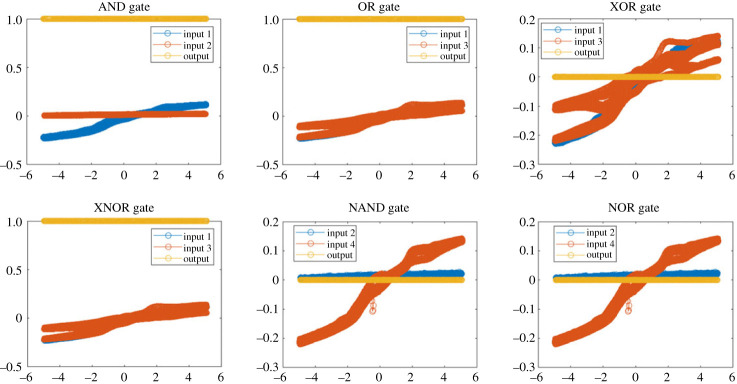
Implementation of fundamental logic operations on proteinoid-microbial biofilm output signals. The applied voltages for the input signals ranged from −4 to +4 V. The output signals that had been processed underwent thresholding to convert them into binary levels. Subsequently, bitwise operators such as AND, OR, XOR, XNOR, NAND and NOR were applied to achieve logical operations. The use of duplicate gates, denoted by subscripts 1 and 2, serves to illustrate the logical operation that is done to distinct output signal pairs originating from diverse biofilm samples. The ability to create logic gates demonstrates the promise of proteinoid-microbial biofilms in the field of biomolecular information processing and computation.

On the output signals derived from ‘Kombucha–proto Brain’, fundamental logical operations were implemented. Samples A-D’s input–output data was processed in Matlab to generate AND, OR, XOR, XNOR, NAND and NOR logic gates (figure 12). The continuous output voltages were initially converted into discrete binary levels with the application of a threshold value of 0.5V. The binary output signals were subsequently subjected to bitwise operations such as AND (&), OR (|) and NOT (∼) in order to execute the logic functions. The construction of the AND gate involved the computation of the bitwise AND operation between Outputs 1 and 2. The NOR gate was derived through the application of bitwise negation (∼) to the bitwise OR operation performed on Outputs 1 and 3. Various combinations of the four sample output signals were employed to evaluate the effectiveness of all logic gates, so demonstrating their broad applicability. The potential of using proteinoid-microbial biofilms for biomolecular computing has been demonstrated by their ability to execute signal processing and logic operations.

The basic logic gates can be represented mathematically using Boolean algebra notations. The AND gate performs a logical conjunction on the inputs, described by

3.2 $$y=x_{1}\wedge x_{2}.$$

The OR gate performs a logical disjunction

3.3 $$y=x_{1}\vee x_{3}.$$

Exclusive OR (XOR) outputs true when the inputs differ

3.4 $$y=x_{1}⊕x_{3}.$$

The XNOR gate is the inverse of the XOR operation

3.5 $$y=x_{1}↔x_{3}.$$

The NAND and NOR gates represent AND and OR with negation. For NAND

3.6 $$y=\neg (x_{2}\wedge x_{4}).$$

And NOR

3.7 $$y=\neg (x_{2}\vee x_{4}),$$

where ∧ is AND, ∨ is OR, ⊕ is XOR, ↔ is XNOR and ¬ is NOT. These equations relate the input variables *x*_1_, *x*_2_, *x*_3_, *x*_4_ to the output *y* for each logic operation.

The implementation of logic operations involved the use of discrete thresholding and bitwise operators on the output signals of the biofilm.

3.8 $$V_{\mathrm{out}}^{\prime }=\left\{ \begin{array}{cc} 1\; & \text{if}\;V_{\mathrm{out}}>0.5\;\text{V}\;\\ 0\; & \text{if}\;V_{\mathrm{out}}\leq 0.5\;\text{V}\; \end{array}\right..$$

The measured output voltage is denoted as *V*_out_, whereas the discretized binary voltage used for logic operations is represented as *V*′_out_.

The use of fundamental logical operations implies that proteinoid-microbial biofilms have the potential to function as biosynthetic materials for unconventional computing. The nonlinearity and structural diversity of these elements allow for intricate signal changes that can be compared to logic operations (table 5).

**Table 5.** A truth table is provided to concisely outline the input–output relationships for fundamental logic operations. The implementation of logic gates involves the use of discrete binary thresholding and mathematical bitwise operators on the output signals of the proteinoid-microbial biofilm. The logical operations of conjunction, disjunction and exclusive disjunction are implemented in computer systems using the bitwise operators &, | and ⊕. These operators perform the respective operations on binary input values [55]. The inherent nonlinearity and compositional diversity of proteinoid-microbial biofilms enable the processing of signals and execution of computational operations, as evidenced by the formation of digital logic patterns.

input 1	input 2	AND	OR	XOR	XNOR	NAND	NOR
0	0	0	0	0	1	1	1
0	1	0	1	1	0	1	0
1	0	0	1	1	0	1	0
1	1	1	1	0	1	0	0

**Figure 13.**
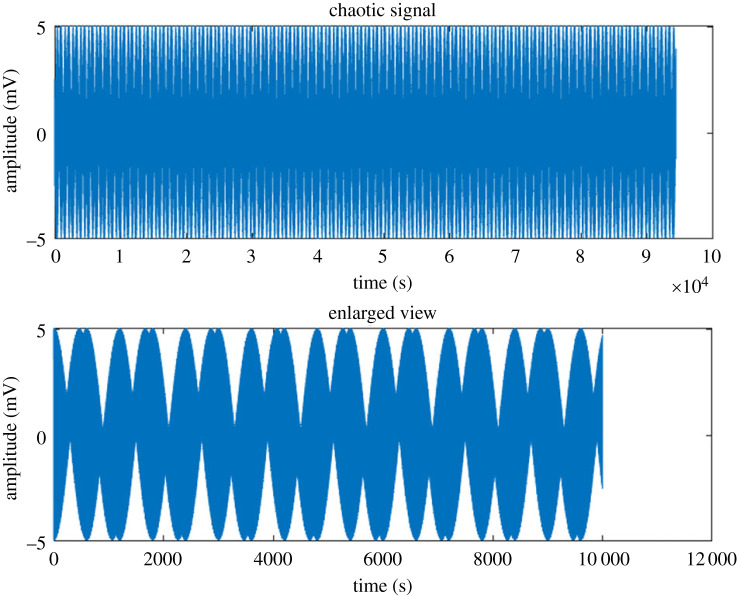
The proteinoid-microbial biofilms produce a chaotic fusion of signals. Chaotic signal synthesis by random switching of input signals. Subplot shows detailed view from 4 − 5 × 10^4^ s. The subplot on enlargement provides additional information about the intricate and rapidly evolving nature of the chaotic signal during that specific time period. The phenomenon of random switching between input signals is associated with the introduction of unpredictability and information encoding, which are characteristic features of chaotic systems. The use of inherent disorder within proteinoid-microbial systems has the potential to facilitate the implementation of chaotic computing methodologies for encryption, resilience to noise and nonlinear information processing. The investigation of various combination schemes and feedback mechanisms has the potential to reveal emergent behaviours in evolving bio-computing architectures that use chaos.

The proteinoid-microbial biofilm samples demonstrated intricate oscillatory signals, which serve as an essential precursor for chaotic dynamics (figure 13). By employing a process of extracting four signals and subsequently subjecting them to a simplistic chaotic mapping technique that merged them in a nonlinear manner, we successfully produced a novel composite signal characterized by randomized transitions between the inputs.

The previously mentioned combination technique brought key characteristics associated with chaos, including unpredictability, information encoding and heightened sensitivity to the initial conditions. Additional quantitative study is necessary in order to comprehensively characterize the chaotic qualities using methodologies such as Lyapunov exponents and fractal dimensions [56–58]. The incorporation of chaotic aspects has the potential to enhance security in communication systems, facilitate disruptive computation and offer fault-tolerance capabilities. The inherent biological intricacy present in these composite systems can be used for the development of bioinspired chaotic processors or reservoirs in the field of machine learning. The implementation of more sophisticated combination methods and recursive feedback loops has the potential to improve the observed chaotic behaviours. In simple terms, the use of proteinoid-microbial biofilms has the potential to offer a significant source of intricate multi-dimensional signals that can be harnessed for unconventional computing techniques by leveraging on the phenomenon of chaos [55,59–61].

### 3.5. Characterizing electrical spikes in Kombucha proto brain

**Figure 14.**
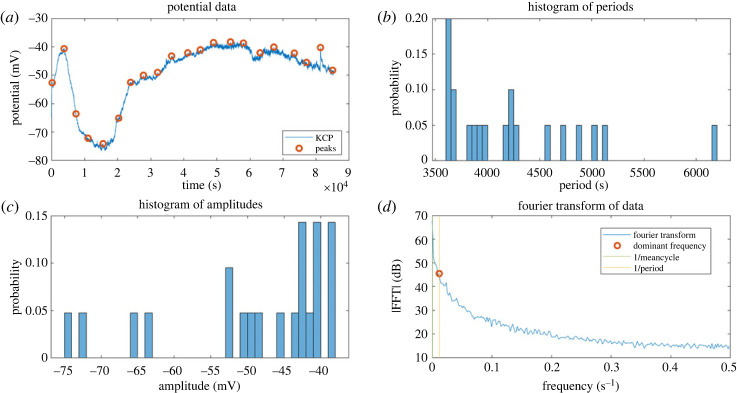
KCP oscillation patterns are statistically analysed. (*a*) Time-lapse voltage trace with rhythmic spikes in mV. (*b*) Histogram of oscillation times in seconds, with a mean of 4240.65 s and a median of 4068.50 s. (*c*) Histogram of spike amplitude peaks in mV, with a mean of −48.58 mV, a median of −43.25 mV and a standard deviation of 11.09 mV. (*d*) Fourier transform examination of the oscillatory signal reveals prominent frequencies. This multipanel image quantifies important periodicity and amplitude measures used to describe proteinoid spiking kinetics.

**Figure 15.**
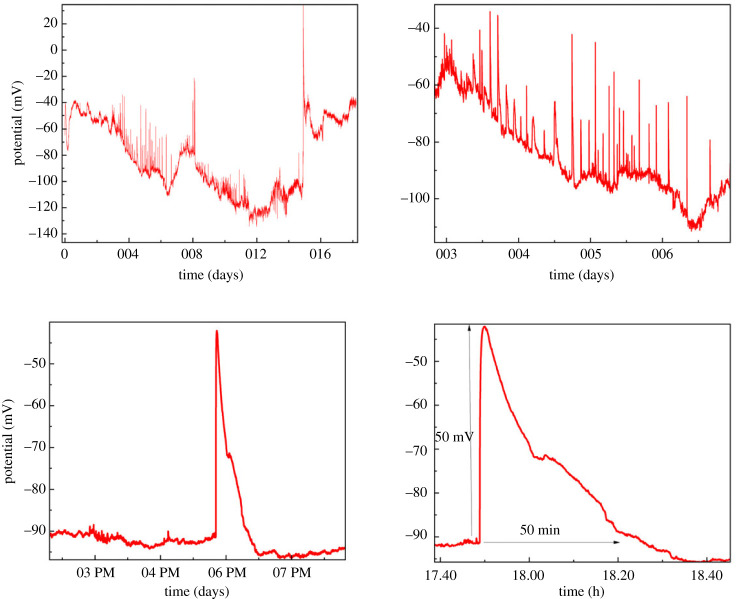
The dynamics of spiking in a KCP proto brain following an abrupt depolarization. Following an initial 50 mV depolarizing pulse disturbance, the voltage trace exhibits repeated spikes over time. The spikes display rapid depolarization upstrokes followed by delayed repolarization downstrokes, with an amplitude of about 50 mV above baseline. The periodicity of the surges is approximately 50 min on average. This activity reflects the proteinoid spiking response to a rapid depolarizing stimulus, revealing the spike shape, amplitude, kinetics and periodicity of oscillating voltages.

**Figure 16.**
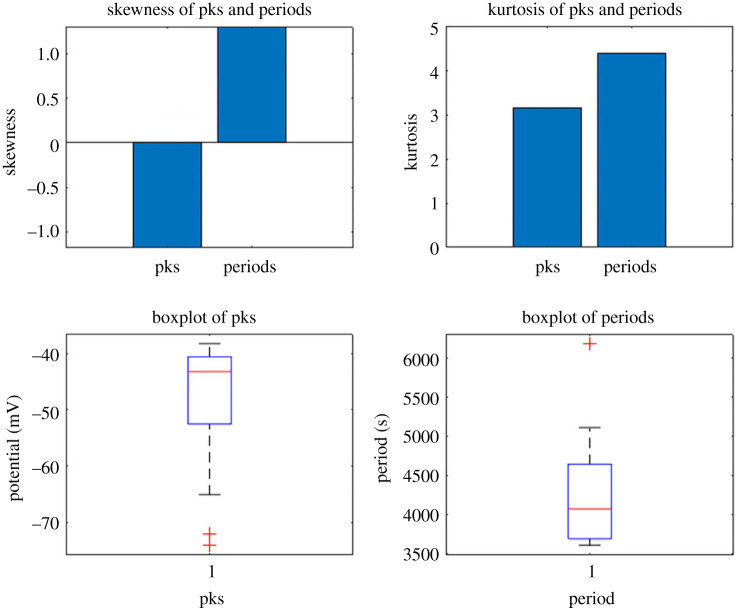
Higher-order statistical characterization of KCP sample spiking parameters. Peak values with a skewness of −1.18 indicate left-sided asymmetry. 3.15 kurtosis indicates a distribution that is near to normal. The positive skewness of 1.30 for periods indicates right-sided asymmetry. The increased kurtosis value of 4.38 indicates a leptokurtic distribution with heavier tails. The boxplots depict the data ranges, quartiles and outliers for the distributions of peak values and periods. This analysis provides a comprehensive multivariate description of the variability in proteinoid spiking peak and periodicity.

Figure 14 provides a robust multivariate description of the oscillation patterns of KCP proto brains. Over time, the voltage trace reveals rhythmic spiking activity. The periodicity analysis reveals a mean surge interval of approximately 4000 s, or 1 h. The amplitude histogram reveals a normal distribution of spike peaks with a mean value of −45 mV. Fourier analysis uncovers the dominant frequencies contributing to the periodic spikes in voltage. Figure 15 illustrates the spiking response to a depolarizing pulse. Rapid depolarization of 50 mV causes repetitive spikes with a regular period of 6 min. The shape of the spike exhibits rapid rise and slower decline kinetics, with an amplitude of 25 mV. This reveals intrinsic oscillatory properties of the KCP proto brain system. Figure 16 further examines the spikes’ higher-order statistics. Data with a left-skewed amplitude distribution indicates a high proportion of negative spike peaks. The leptokurtic periods suggest a distribution with heavy tails, with a high probability of extremely long and brief intervals. This demonstrates the variability and complexity of KCP spiking patterns.The results offer valuable insights into the emerging functionality of synthetic proto-brains made up of basic proteinoid components and Kombucha living skin. The KCP nanostructures exhibit neuron–like signalling behaviours, as evidenced by the observed voltage oscillations, spike statistics and response to perturbations. The rhythmic patterns, characterized by millisecond spike kinetics and periodicities ranging from minutes to hours, exhibit intrinsic excitability that is reminiscent of primitive neural networks. The dynamics exhibit complexity, as evidenced by the normal amplitude distributions and asymmetric periodicity statistics. The ability to regulate the activity of proteinoid spiking through stimuli such as rapid depolarization indicates the potential for transmitting information. By tuning parameters such as spike intervals and patterns, it becomes possible to encode signals.The combination of quantitative characterization and excitation of synthetic proto-brain dynamics holds great potential for advancing our understanding of the origins of cognition. Further investigation into the physical factors that influence proteinoid self-assembly and the formation of nanostructures could reveal principles for designing customized synthetic neural circuits.

### 3.6. Tracking electrical memory effects in microbial-proteinoid composites via dynamic I–V curves

**Figure 17.**
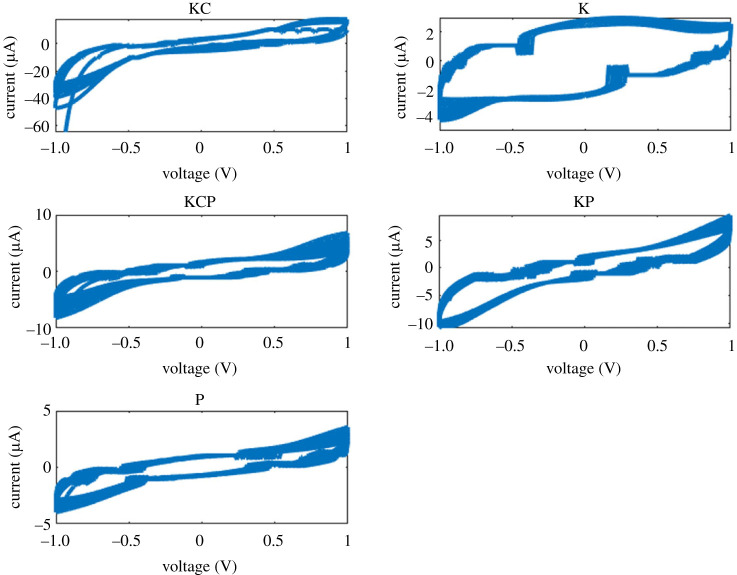
Current–voltage (I–V) profiles for Kombucha-Chlorella (KC), Kombucha (K), Kombucha-Chlorella-proteinoid (KPC), Kombucha-proteinoid (KP) and proteinoid (P) samples. The I–V curves indicate pinched hysteresis loops that are characteristic of memristive systems. Among them, KC demonstrates the broadest current range, spanning from −60 to 1 μA. K demonstrates a narrower semi-circular shape from −4 to 2 μA, while KPC, KP and P show similar intermediate current ranges on the order of −10 to 7 μA. The composites including proteinoids (KPC, KP and P) exhibit a high degree of resemblance to the ideal behaviour of memristors, as indicated by the extent of pinching observed. The dynamics observed in this figure demonstrate the modification of conductance dependent on voltage, which is a result of the interconnected networks between proteinoid and microbial networks.

Distinct pinched hysteresis loops, which are characteristic of memristive systems, were observed in the current–voltage characterization of the composite samples (figure 17). The I–V curves exhibited by the proteinoid-containing biofilms (KPC, KP and P) have a strong similarity to the ideal memristor behaviours, as evidenced by the extent of pinching seen. On the other hand, the samples containing simply microbes, namely K and KC, displayed memristive properties that were less prominent. However, the observation of electrical memory effects in all the mixed proteinoid-microbial compositions indicates the existence of cooperative relationships within the hybrid biofilms. These interactions facilitate the regulation of conductivity, which is dependent upon ‘remembering’ of the applied voltage bias [62].

**Figure 18.**
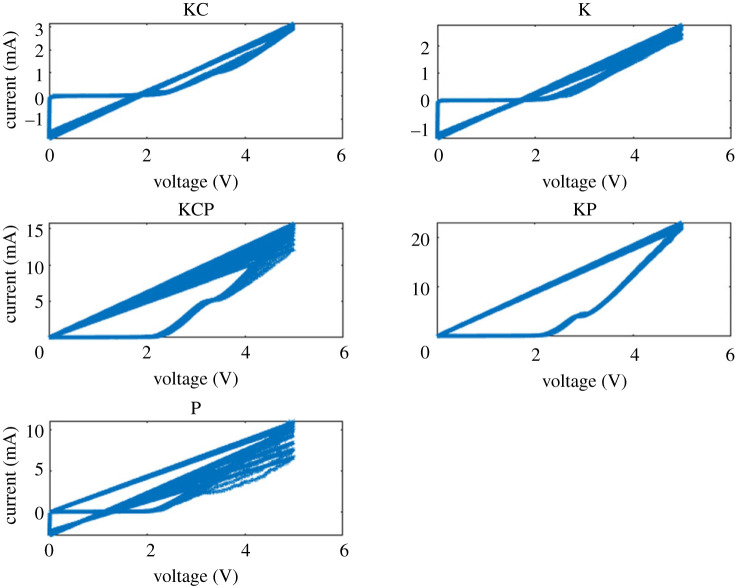
The current–voltage profiles of the Kombucha-Chlorella (KC), Kombucha (K), Kombucha-Chlorella-proteinoid (KCP), Kombucha-proteinoid (KP) and proteinoid (P) samples were monitored over a span of 20 cycles, ranging from 0 to 5 V. The composites KPC, KP and P, which are rich in proteinoids, demonstrate significantly higher maximum current values of 15 mA, 20 mA and 10 mA correspondingly. By contrast, the highest current values for KC and K are approximately 3 mA. Pronounced pinched hysteresis loops are seen in the KPC, KP samples, suggesting amplification in their memristive characteristics. By contrast, the I–V curves of KC, K and P do not exhibit memristive switching behaviours. The presence of proteinoid components facilitates significantly enhanced conductivities and notable electrical memory effects, which have great potential for the development of neurorobotic interfaces.

The analysis of the composite biofilms’ current–voltage relationship demonstrated significant differences between the networks comprising proteinoids and those consisting only of microbes, as depicted in figure 18. During multiple voltage cycles, the KPC, KP and P samples exhibited habituation, characterized by a decrease in hysteresis, as well as memory, demonstrated by a return to a heightened conduction state after stimulation. On the other hand, KC and K demonstrated crossing I–V curves that were devoid of any memory effects. The observed I–V signals demonstrate a similarity between the forgetting characteristics of KC/K and the memory stability observed in the proteinoid-integrated biofilms. The proteinoids have a significant capacity for flexibility and adaptive conductivity, enabling the composite system to retain and encode information from previous inputs.

The proteinoid-microbial composites demonstrate dynamic electrical behaviours that indicate the presence of various adaptive molecular processes responsible for their learning and memory features (table 6). The observed hysteresis and memristive switching exhibit significant characteristics, which serve as evidence for voltage-mediated transitions between meta-stable conductive states. These transitions have the potential to facilitate transitory information storage. In the context of periodic stimulus response, the phenomenon of progressive habituation indicates that factors such as ion depletion may play a significant role. The capacity to computationally learn and remember past inputs is critical for engineering intelligent biosystems. The current results showcase the presence of important adaptive features in easily adjustable synthetic biofilms. These features are made possible through the combined interactions of biological components.

**Table 6.** Proposed adaptive mechanisms in proteinoid-microbial biofilms.

phenomena	potential molecular mechanisms
learning	the conformation and alignment of proteinoids undergo modifications in response to voltage stimulation, resulting in the alteration of conductivity channels and junctions between the various components [63–65]
remembering	meta-stable high conduction states can be achieved by applying voltages and are sustained through cooperative interactions and regenerative feedback mechanisms [66,67]
habituation	the process of repeated stimulation leads to the depletion of mobile ions and induces conformational relaxation, resulting in a decrease in hysteresis while maintaining its initial conductivity [62,68,69]

## 4. Discussion

This study showcases the potential of composite KP biofilms as adaptive and electroactive biosystems for interacting with neural cells, as depicted in the mind map illustration (figure 20). This type of effect encompasses memristive switching, intrinsic spiking patterns and dynamic conductive responses.

The Kombucha-proteinoid biofilms include memristive switching characteristics that enable them to replicate the plasticity observed in biological synapses [70]. Similar to the way neurons adjust their connections in response to signals, these biofilms have the ability to regulate their conductivity by responding to voltage inputs. This gives them the ability to learn and store information over time. The spiking patterns relate to oscillations in voltage or current that have the ability to propagate across the biofilm, like action potentials observed in axons [37,71,72]. The dynamic electrical characterizations showed significant current fluctuations, spiking patterns and memristive hysteresis loops. These characteristics closely resemble the excitability and plasticity observed in biological neural networks.

**Figure 19.**
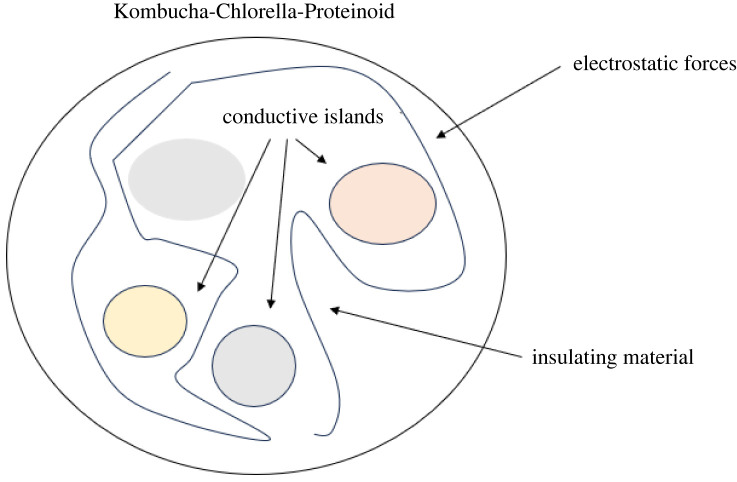
Granular structural model of Kombucha-Chlorella-Proteinoids sample. The large circle on the outside represents the Kombucha growing media. The cells produce proteinoids, which are shown as small coloured circles. Proteinoids are believed to create tiny conductive regions, and the dashed lines indicate the electrical connections between them. This model illustrates a granular semiconductor-like structure formed by proteinoids in the Kombucha-Chlorella system.

Our findings show that Kombucha-Chlorella samples had increased conductivity, indicating semi-conducting behaviour. To explain doping effects in organic semiconducting materials, a granular model consisting of conductive areas linked by electrostatic forces was previously proposed (figure 19). This model may provide insights into the conductive mechanism observed in the KCP system. Electrical connections between these proteanoid-rich domains could potentially facilitate electron transit through the material, explaining the observed increase in conductivity [73].

Semiconductors are made up of small crystal grains packed together. In between these grains are regions called inter-grain boundaries. Here, the electrical properties change suddenly compared to inside the grains. This is because the charge density and band structure differs at these boundaries.

The inter-grain boundaries in the semiconductor can function as obstacles or barriers that impede the flow of electrical charges. The ability to block or trap charges is dependent upon factors such as the applied voltage, temperature and the presence of any magnetic field.

There are several factors that determine how charges can move past the inter-grain boundaries. These include the size and shape of the grains, which way they are oriented, what they are made of, and how much surface area they share. The charges have to find ways to get around or jump over these boundaries between the grains [74].

The adaptive and electroactive properties of KP biofilms have potential implications in the field of neuromorphic computing, alongside their existing applications in medical and engineering fields. The cutting-edge approach aims to replicate the structure and functions of biological neural networks using synthetic materials and devices. The behaviours exhibited by these biofilms, such as conductivity modulation, plasticity and spiking activity, closely resemble the necessary characteristics for implementing artificial/biological neural networks in hardware.

This work sets the groundwork for engineering intelligent biosystems that can computationally mimic the brain’s adaptive abilities in the future. The investigation of the precise interactions between proteinoids and microbes that influence the magnitude of conductivity and spiking dynamics has the potential to provide insights into ways for enhancing performance. The integration of additional nanoscale modifiers has the potential to reveal novel hybrid films that possess enhanced bioelectric capability [75–77].

In the realm of computer, the use of external stimulus patterns to train synthetic biofilms presents an opportunity for the integration of machine learning, enabling unconventional, biologically inspired information processing. In general, this study emphasizes potential avenues for integrating biological elements in order to develop intelligent biosystems that incorporate adaptivity, memory and problem-solving abilities. By delving into intricate and multi-faceted designs, as well as investigating the transmission of signals inside interconnected networks, researchers may reveal novel functionalities for unconventional computing systems inspired by biological processes [78].

In contrast to conventional metal electrodes, the KP biofilms exhibit enhanced biocompatibility and possess the ability to establish conformal contact with neural tissue. In relation to inflexible nanomaterials, biofilms have a malleable and organ-like texture. Nevertheless, there are still obstacles to overcome in relation to the ability to scale up and ensure the long-term durability of Kombucha-based biosystems prior to their extensive use in medical implants or bioelectronic applications.

Finally, integrating Kombucha biofilms with conventional semiconductor technology and programming their connectivity in an organized manner requires significant research. It will be crucial to carefully adjust the biofilm thickness, architecture, conductivity and spike patterns in order to effectively train and optimize their performance. If successfully developed, neuromorphic platforms based on Kombucha could offer more natural and efficient computing paradigms in comparison to traditional von Neumann architectures. This application showcases a thrilling new path for using these adaptive biosystems beyond the field of biomedicine.

**Figure 20.**
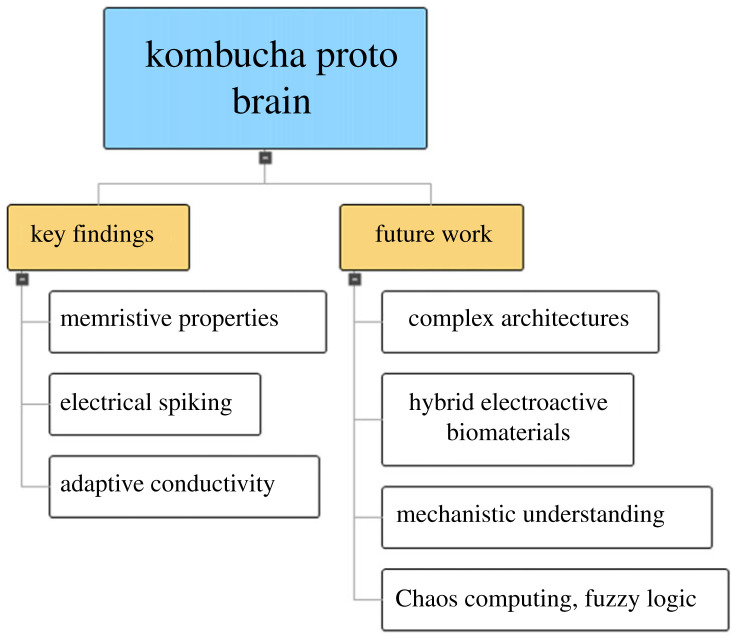
Key research findings highlighted include the emergent memristive properties, electrical spiking behaviours and adaptive conductivity exhibited by the Kombucha–proto brain. Several future research directions can be proposed, including the optimization of composition, the development of more complex architectures, the acquisition of mechanistic understanding, and the exploration of applications in unconventional computing.

The potential of KP biofilms for interacting with neural cells and neuromorphic computers is emphasized in the suggested applications, indicating the broad range of possibilities for these versatile biosystems beyond the field of biomedicine (figure 20). However, it is imperative that these biofilms satisfy a number of characteristics in order to successfully supplant traditional computing architectures, as outlined in table 7. The key factors encompass the attainment of programmable connectivity and plasticity, the ability to adjust spiking patterns, the capacity for learning, biocompatibility, scalability, long-term stability and uniform interface with electronic systems. To optimize these biosystems as biological neural networks for computation, it will be necessary to make significant advancements in other areas. The development of a mechanistic understanding of the electroactive characteristics and behaviours that arise from the hierarchical organization and symbiotic interactions of bacteria, yeasts and proteins within Kombucha biofilms presents both opportunities and challenges that are closely interconnected. Current research endeavours aim to uncover the complete capabilities of these living materials in order to advance bioelectronic technology for future generations.

**Table 7.** The essential criteria and conditions for using Kombucha-proteinoid biofilms as an alternative for conventional computing systems.

criteria	necessary conditions
connectivity	the ability to programme synaptic conductivity with a high number of connections per neuron
plasticity	dynamic modulation of synapse strength through memristive switching
spike patterns	action potential spikes and tunable oscillations
‘Learning’	adaptive alterations in connectivity and conductivity in response to stimuli
biocompatibility	biodegradable, non-toxic materials and interfaces
scalability	architecture hierarchical across multiple dimension scales
stability	robust electroactive performance over the course of several months/years
integration	uniform interfaces between microorganisms and electronic components

## 5. Conclusion

In summary, this study showcases the potential of composite KP biofilms as versatile bioelectronic materials for connecting with neuronal networks. The proteinoid nanonetworks and Kombucha polysaccharide matrix collaborate to form a unified system that demonstrates emergent electrical excitability, resembling important characteristics observed in brain tissues found in nature. The proteinoid-Kombucha films exhibit dynamic electrical spikes, tunable conduction and intrinsic habituation, which contribute to their ability to mimic the signalling activities of biological neurons. This research offers a framework that combines the processability of biomolecular materials with the complexity of living systems.

## Data Availability

These data are accessible via the online database Zenondo https://doi.org/10.5281/zenodo.10037497 [79]. Supplementary material is available online [80].
